# The why and how of the SynNerGe criteria of Parkinson´s disease

**DOI:** 10.1007/s00702-024-02797-9

**Published:** 2024-06-25

**Authors:** Günter U. Höglinger, Anthony E. Lang

**Affiliations:** 1 Department of Neurology with Friedrich Baur Institute, LMU University Hospital, Ludwig-Maximilians-University (LMU) Munich, Marchioninistr. 15, 81377 Munich, Germany; 2 German Center for Neurodegenerative Diseases (DZNE), Munich, Germany; 3 Munich Cluster for Systems Neurology (SyNergy), Munich, Germany; 4 Edmond J. Safra Program in Parkinson’s Disease and the Rossy PSP Centre, Toronto Western Hospital, University of Toronto, Toronto, ON Canada

**Keywords:** Biological definition, Genetics, Synuclein, Neurodegeneration, Parkinson’s disease

## Abstract

In pursuit of early therapeutic interventions for Parkinson’s disease, the proposed SynNeurGe classification system integrates α-synuclein pathology (S), neurodegeneration evidence (N), and pathogenic gene variants (G). This approach aims to address the disease’s complexity and biological diversity. It suggests categorizing patients based on the presence or absence of α-synuclein pathology in tissues or cerebrospinal fluid, neurodegeneration indicators from specific imaging techniques, and identification of pathogenic gene variants associated with Parkinson’s disease. The proposed system emphasizes the future need for precision medicine and aims to facilitate both basic and clinical research toward disease-modifying therapies. However, the authors stress that initial implementation should be confined to research settings, considering ethical implications and current limitations. Prospective validation of these criteria is deemed necessary to ensure their efficacy and ethical application in clinical practice.

## Introduction

Parkinson’s disease (PD) research has made significant strides in understanding the molecular causes and pathogenesis of the disorder, opening avenues for the development of impactful disease-modifying therapies. This progress underscores the recognition that PD, traditionally viewed as a clinicopathologic entity, may originate from diverse genetic or environmental triggers acting through partially overlapping pathways. Neuropathological studies have spotlighted Lewy pathology and misfolded α-synuclein as pivotal in PD pathophysiology, delineating PD from other synucleinopathies while challenging the boundaries between PD and dementia with Lewy bodies.

Advancements in biomarkers — fluid, tissue, and imaging — permit the objective identification of genetic risks, pathological processes, and neurodegeneration even preceding clinical symptoms. Nevertheless, diagnostic criteria for PD rely almost exclusively on clinical features, often late in the disease course and lacking a unified biological framework. To address this, a proposed biological classification for research, termed SynNeurGe (Höglinger et al. 2024), integrates pathological α-synuclein presence (S), neurodegeneration markers (N), and genetic factors (G). This approach aims to enhance preclinical and clinical diagnosis, allowing stratification based on active pathogenic mechanisms.

The proposal recognizes the presence of PD-related biological changes long before clinical onset, emphasizing the potential for early detection and intervention. However, its application remains exclusive to research, necessitating future studies to ascertain its predictive value in individuals yet to manifest clinical symptoms. This shift towards a biological definition of PD acknowledges the disease’s complex trajectory and provides a framework for more precise diagnosis, staging, and therapeutic interventions, essential for advancing PD research across various domains including epidemiology, pathogenesis, biomarker discovery, clinical trials and eventually precision medicine.

In summary, the proposed SynNeurGe classification offers a promising avenue for refining PD diagnosis and understanding its underlying biology, thus paving the way for more effective disease-modifying treatments. However, further research is crucial to validate its utility and to explore its implications for clinical practice.

## Synucleinopathy

The pathology of PD is characterized in most but importantly, not all instances by the presence of aggregated α-synuclein as Lewy bodies and Lewy neurites in the nervous system. Pathological forms of misfolded α-synuclein are believed to play a crucial role in the disease’s development and progression. Biomarker development has progressed significantly in recent years, allowing the designation of Parkinson’s type synucleinopathy status in living patients.

The proposal suggests classifying individuals as α-synuclein-positive (S+) if specific pathological tests confirm the presence of α-synuclein and α-synuclein-negative (S-) otherwise (Table 1). Pathological α-synuclein species should be a defining molecular anchor for PD classification. The proposed biological classification acknowledges asymptomatic S + individuals as having Parkinson’s type (or Lewy-type) synucleinopathy, even though their progression to clinical disease remains uncertain to date. Although yet to be proven through future research, such individuals might be expected to harbor the pathology of “incidental Lewy body disease”.

**Table 1:** Table 1

Designation	Abnormality	Characteristics*
**Parkinson’s/Lewy type synucleinopathy**		
S^+^	α-syn SAA in CSF	Sensitivity high / Specificity high
S^+^	α-syn SAA in skin	Sensitivity high/ Specificity high
S^+^	α-syn IHC/IHF in skin	Sensitivity moderate / Specificity high
Exclusion criterion ruling out S^+^	Elevated Neurofilament Light chain (NfL)	Sensitivity high for atypical parkinsonism / Specificity high for MSA
Exclusion criterion ruling out S^+^	Neuroimaging features of MSA (e.g., characteristic changes in the putamen, cerebellum and pons)	Sensitivity moderate / Specificity high
**PD-associated neurodegeneration**		
N^+^	DAergic PET/SPECT (Striatal dopaminergic deficit)	Sensitivity high / Specificity low
N^+^	Metabolic FDG PET (PD related brain metabolic pattern)	Sensitivity high / Specificity high
N^+^	Cardiac MIBG SPECT (Sympathetic cardiac denervation)	Sensitivity moderate to high/ Specificity moderate
Exclusion criterion ruling out N^+^	Structural MRI (Findings characteristic of atypical parkinsonism)	Sensitivity moderate / Specificity high
Exclusion criterion ruling out N^+^	FDG PET (Findings characteristic of atypical parkinsonism)	Sensitivity high / Specificity high
**PD-specific pathogenic gene variants**		
G_F_^**+**^	*SNCA* monoallelic triplication	Fully penetrant / Parkinson’s type synucleinopathy
G_F_^**+**^	*SNCA* monoallelic pathogenic single nucleotide variants	Fully penetrant / Parkinson’s type synucleinopathy
G_F_^**+**^	*PRKN* biallelic pathogenic variants	Fully penetrant / in ~ 20% of the cases only Parkinson’s type synucleinopathy
G_F_^**+**^	*PINK1* biallelic pathogenic variants	Fully penetrant / uncertain association with Parkinson’s type synucleinopathy
G_F_^**+**^	*PARK7* biallelic pathogenic variants	Fully penetrant / uncertain association with Parkinson’s type synucleinopathy
G_P_^**+**^	*SNCA* monoallelic duplication	Strong predisposition / Parkinson’s type synucleinopathy
G_P_^**+**^	*LRRK2* monoallelic (or biallelic) pathogenic variants	Strong predisposition / in most cases Parkinson’s type synucleinopathy
G_P_^**+**^	*VPS35* monoallelic pathogenic variants	Strong predisposition/ uncertain association with Parkinson’s type synucleinopathy
G_P_^**+**^	*CHCHD2* monoallelic pathogenic variants	Strong predisposition / uncertain association with Parkinson’s type synucleinopathy
G_P_^**+**^	*GBA1* monoallelic severely pathogenic variants	Medium predisposition / Parkinson’s type synucleinopathy

*high > 80%; moderate > 70 < 80%; low < 70%. G_F_^+^: fully penetrant pathogenic gene variants, G_P_^+^: pathogenic gene variants with strong or intermediate predisposition. α-syn: α-synuclein; CBS: corticobasal syndrome; CSF: cerebrospinal fluid; DAergic: dopaminergic; FDG: fluoro-deoxy-glucose; IHC: immunohistochemistry; IHF: immunohistofluorescence; MIBG: metaiodbenzylguanidin; MRI: magnetic resonance imaging; MSA: multiple system atrophy; MSA: multiple system atrophy; PD: Parkinson’s disease; PD: Parkinson’s disease; PET: positron emission tomography; PSP: progressive supranuclear palsy; PSP: progressive supranuclear palsy; SAA: seeding amplification assay; SPECT: single-photon emission computerized tomography

Various methods have been explored to detect pathological α-synuclein in biological biofluids, foremost CSF, and various tissues. Skin biopsies have shown high promise in distinguishing PD from other conditions. The development of α-synuclein seed amplification assays has revolutionized the potential for a widespread biological diagnosis of PD, with high sensitivity in multiple biological samples, particularly CSF and skin. However, caveats exist, particularly in differentiating PD from multiple system atrophy, which need to be addressed by additional exclusionary examinations. Advances in blood-based assay techniques are expected to enhance diagnostic capabilities in the future.

Numerous other biological pathways are implicated in PD, leading to the evaluation of candidate biomarkers. However, none reliably distinguish PD from controls or other parkinsonian disorders so far, due to biological heterogeneity and technological limitations. Therefore, the S + or S- component of the proposed biological classification should rely on validated assays in skin biopsies or CSF, with ongoing investigation into other tissues, fluids, and methods. We emphasize that S- individuals may still qualify for a diagnosis of PD in case they harbor a pathogenic gene variant which does predispose for PD without associated synucleinopathy.

In summary, the proposal advocates for a biological classification of PD based on the presence or absence of Parkinson’s/Lewy type synucleinopathy, utilizing validated assays to document α-synuclein pathology (see Table 1). While advancements in biomarker development hold promise for improving PD diagnosis and classification, further research and validation are needed to ensure reliability and accuracy in clinical practice.

### Neurodegeneration

The definition of neurodegeneration in biologically defined PD relies on several key findings (Table 1), although current methods predominantly focus on nigrostriatal dopamine projection and have therefore limited specificity in distinguishing PD from other neurodegenerative parkinsonian disorders.

Dopaminergic denervation, a principal confirmation of PD-associated neurodegeneration, is detected through reduced striatal uptake observed with molecular imaging markers for dopamine transporter, vesicular monoamine transporter 2, or aromatic amino acid decarboxylase. However, similar findings are also present in multiple system atrophy and progressive supranuclear palsy, limiting specificity.

Another indication of PD-associated neurodegeneration is altered glucose metabolism, evidenced by [18 F]fluorodeoxyglucose PET. Changes in glucose metabolic networks, known as Parkinson’s disease-related pattern, indirectly reflect nigrostriatal dopaminergic neuron loss, providing presumptive evidence of pre-synaptic denervation with intact post-synaptic basal ganglia connections., Similar changes occur in prodromal disease, such as REM-sleep behavior disorder but also with the use of dopamine receptor blocking drugs. Specificity of [18 F]fluorodeoxyglucose pattern within degenerative parkinsonism forms is high due to different characteristic patterns in atypical parkinsonisms.

Additionally, cardiac sympathetic denervation, evidenced by reduced tracer uptake on meta-iodobenzylguanidine SPECT (also with F-dopamine PET), indicates PD-associated neurodegeneration. While specificity of cardiac sympathetic imaging is high, it is imperfect, as abnormalities have been reported in individuals affected by other conditions including progressive supranuclear palsy and multiple system atrophy.

Non-dopaminergic molecular imaging of other neurotransmitter systems, such as serotonin, noradrenaline, and acetylcholine, remains under investigation and lacks validation for defining PD-related neurodegeneration. Similarly, imaging techniques like iron-sensitive MRI and neuromelanin imaging show promise as potential markers but are still considered investigational.

The recommendation is to classify individuals as neurodegeneration-positive (N+) if specified pathological tests confirm PD-associated neurodegeneration (Table 1), with all other conditions considered as neurodegeneration-negative (N-). Despite recent advances, the lack of specificity in current methods underscores the need for further research and validation to enhance the accuracy of neurodegeneration diagnosis in PD.

### Genetics

To date, up to 15% of PD patients carry a monogenic pathogenic variant, with certain populations, such as Arab Berbers, exhibiting even higher rates of up to 40%. Confirmed monogenic forms of PD include dominantly inherited forms (*SNCA, LRRK2, VPS35, and CHCHD2*) and recessively inherited forms (*PRKN, PINK1, and PARK7*). The likelihood of developing clinical PD in asymptomatic carriers of a pathogenic variant varies depending on the gene involved and the specific variant. For instance, only certain variants within *GBA1* significantly increase the risk of manifesting PD with reduced penetrance, qualifying them for use in proposed biological classifications.

Different levels of pathogenic effects are proposed. The first level encompasses fully penetrant variants (G_F_^+^) like *SNCA* triplications, *SNCA* missense variants, and biallelic *PRKN*, *PINK1*, and *PARK7* missense, nonsense, small indels, and copy number variants. The second level includes variants conferring strong or intermediate predisposition to PD with incomplete penetrance (G_P_^+^), including *SNCA* duplications and pathogenic variants in *LRRK2*, *VPS35*, *CHCHD2*, or *GBA1*.

The degree of predisposition for Parkinson’s/Lewy type synucleinopathy also varies among specific pathogenic gene variants. For instance, variants in *SNCA* and *GBA1* unequivocally predispose to Parkinson’s/Lewy type synucleinopathy. *LRRK2* monoallelic or biallelic pathogenic variants usually predispose to synucleinopathy, although cases of neurodegeneration without synucleinopathy exist. Biallelic variants in *PRKN* predispose to synucleinopathy in approximately 20% of cases.

The recommendation is to report a person’s PD genetic status as positive if they carry a fully penetrant pathogenic variant or a pathogenic variant with strong or intermediate predisposition (Table 1). All other conditions, such as low predisposition pathogenic gene variants or polygenic risk scores, are considered genetically indeterminate.

In summary, understanding the genetic basis of PD is crucial for diagnosis and prognosis. By categorizing pathogenic variants based on their predisposition to PD and Parkinson’s/Lewy type synucleinopathy, clinicians can better assess disease risk and tailor treatment strategies accordingly. Ongoing research will continue to refine our understanding of the genetic landscape of PD and its implications for clinical practice.

### Biological classification

The biological classifications of sporadic and genetic PD, delineated by various combinations of biomarkers, are essential for accurate diagnosis. However, it is crucial to recognize the potential for false negative findings in the categories of pathological α-synuclein (S), neurodegeneration (N), and genetic predisposition (G) due to current technical limitations.

An isolated S + designation characterizes Parkinson’s/Lewy type synucleinopathy when N^+^ is not yet confirmed. In individuals without known genetic predispositions (G), an S ^+^ designation is a prerequisite for classifying sporadic PD biologically, however, further evidence of N^+^ is necessary, since biomarkers for neuronal dysfunction preceding neurodegeneration are currently not established.

Genetic causes of PD exhibit variable associations with Parkinson’s/Lewy type synucleinopathy. While certain gene variants (e.g., *SNCA*) typically lead to S^+^ as the disease progresses, others (e.g., most *PRKN* variants) may never manifest S+. Hence, individuals with confirmed genetic predispositions (G^+^) may be classified as having PD even if they lack pathological α-synuclein (S^-^), provided the specific gene variant doesn’t invariably lead to Parkinson’s/Lewy type synucleinopathy.

The presence of N + generally indicates the transition from Parkinson’s/Lewy type synucleinopathy to biologically defined PD; thus, we do not consider being S^+^ in isolation sufficient at this time to designate an individual as having a “disease”.

Given the protracted preclinical periods of monogenic conditions, commencing as early as birth or even conception, the classification of hereditary neurodegenerative diseases is evolving to recognize this phase as the earliest stage of the disease. Therefore, individuals designated as carrying fully penetrant pathogenic variants (G_F_^+^) automatically qualify for a diagnosis of genetic PD. Pathogenic gene variants with reduced penetrance (G_P_^+^) are considered predisposing to PD genetically but necessitate additional evidence of neurodegeneration for diagnosis. Pathogenic gene variants with low predisposition, polygenic risk scores, or unknown genetic contributions are regarded as genetically indeterminate within the current classification framework.

In summary, the biological classifications of PD must be approached cautiously, considering the limitations of current biomarkers. As our understanding of the disease evolves, these classifications will likely undergo refinement to improve diagnostic accuracy and guide personalized treatment strategies.

### Clinical manifestations

The categorization of individuals as S^+^ or G^+^ necessitates further subdivision based on their clinical status, irrespective of their N status, as signs and symptoms may arise from neuronal dysfunction preceding neurodegeneration or from neurodegeneration in regions that are not assessable by current methods of evaluation. Potentially associated clinical symptoms or signs (C+) are documented, and criteria are applied to establish if they can be attributed to biologically defined PD in affected individuals. These clinical criteria are proposed to be applied to any individual designated as S^+^, N^+^, or G^+^.

Four key considerations define the concept of a C^+^ state. Firstly, early clinical symptoms of PD are diverse and often predominantly non-motor, reflecting pathology outside of brain areas defining clinical PD. There is no uniform order of appearance, precluding definition of a specific non-motor then motor staging. Secondly, clinical symptoms vary in specificity; some are almost pathognomonic, while others remain non-specific, even after diagnosing biological PD. Thirdly, many clinical features are early phase markers of other synucleinopathies, making it challenging to reliably distinguish between these conditions based solely on clinical markers. Finally, the C^+^ state encompasses all clinical stages of disease without distinction between prodromal and later defined disease stages. Further, this approach dose not distinguish between PD and Dementia with Lewy bodies but combines the two for the purposes of biological classification.

The methodology for diagnosing the C^+^ state suggests reporting clinical status in a three-component system: asymptomatic individuals (C^-^), and the presence of defined clinical features possibly (C_poss_^+^) or probably (C_prob_^+^) related to PD. Criteria for the C^+^ states are provided, with each clinical feature presumed to have no other, more probable explanation according to best clinical judgement. Additionally, the development of the feature should be consistent with early PD.

These criteria are to be applied to individuals with biological evidence of PD (S^+^, N^+^, or G^+^). For those without evidence, the International Parkinson and Movement Disorder Society’s (MDS) prodromal Parkinson’s disease criteria (Berg et al. 2015) should be used for individuals without parkinsonism, while the MDS clinical Parkinson’s disease criteria (Postuma et al. 2015) should be used for those with parkinsonism. This comprehensive approach aims to standardize the assessment of clinical symptoms and signs, aiding in the accurate diagnosis and management of PD across different stages of the disease.

## Discussion

The proposed biological classification of PD aims to revolutionize research approaches by categorizing the disease into three key components: Parkinson’s/Lewy type synucleinopathy (S), Parkinson’s disease-associated neurodegeneration (N), and Parkinson’s disease-specific pathogenic gene variants (G). This classification, initially intended for research purposes, addresses the growing need to shift from clinically-based diagnostic approaches to focusing on the underlying biological mechanisms of the disease.

Advances in biomarker development, particularly the ability to detect α-synuclein pathology i*n vivo*, have paved the way for this biological classification. It is envisioned as a framework for future research studies, enabling the implementation of precision medicine approaches for disease modification. Similar biological classifications have been proposed for other neurodegenerative diseases like Alzheimer’s disease (Jack et al. 2016) and Huntington’s disease (Tabrizi et al. 2022), contributing to ongoing research advancements in those fields.

The proposed classification, also denoted as SNG, is comparable to the ATN classification used for Alzheimer’s disease, albeit with distinct differences. While the ATN system does not specify clinical disease status, the SNG approach includes a clinical component, layered onto the binary SNG components. Furthermore, the SNG approach implies an order to the three components, with S^+^ preceding N^+^ in sporadic disease and G^+^ preceding S ^+^ or S^-^ in genetic subtypes. However, it acknowledges that this sequence might not hold true in all cases.

An essential aspect of the proposed classification is the inclusion of an S^-^ designation, recognizing that α-synuclein pathology is not necessary for the development of clinical Parkinson’s disease. This distinction is crucial, as it accounts for the biological heterogeneity of the disease and advances our understanding of its pathology and pathogenesis.

The proposed methodology for diagnosing the clinical status (C^+^) involves a three-component system: asymptomatic individuals (C^-^), and the presence of defined clinical features possibly (C_poss_^+^) or probably (C_prob_^+^) related to Parkinson’s disease. These criteria are applied to individuals with biological evidence of Parkinson’s disease (S^+^, N^+^, or G^+^), facilitating accurate diagnosis and management across different disease stages.

While similar to other biological classifications, such as those for Alzheimer’s and Huntington’s diseases, the proposed classification for PD presents unique features tailored to the complexities of this condition. It provides a comprehensive framework for future research endeavors, aiming to advance science on various fronts, including epidemiology, natural history, neuroimaging, biomarker development, and clinical trials.

Despite its potential benefits, the proposed classification has limitations and concerns, particularly regarding the genetic component. Continuous advancements in understanding genetic and environmental risk factors are expected, necessitating future revisions to incorporate new findings. Ethical concerns also need to be addressed with respect to the implications of this disease classification to asymptomatic S^+^ individuals. Additionally, further studies are needed to validate the proposed biomarkers and optimize testing methods.

In conclusion, the proposed biological classification of PD represents a significant step toward a more nuanced understanding of the disease and lays the foundation for future research endeavors aimed at developing effective disease-modifying therapies and personalized treatment approaches.
